# Supplementary material for: Complexome profiling of the Chlamydomonas psb28 mutant reveals TEF5 as an early PSII assembly factor
Source: Plant Cell. 2025 Mar 17;37(6):koaf055. doi: 10.1093/plcell/koaf055 (PMC12164590; doi:10.1093/plcell/koaf055)
Supplement: koaf055_Supplementary_Data [file koaf055_supplementary_data.zip › psb28_interactive.zip!/plots/intensity/qualityPlots/peptides/CAT1_Cre09.g417150.t1.2.html]

### Cre09.g417150.t1.2

Trivial name: CAT1  
SeqCoverage [median]: 46.20  
Mass: 56.16 kDa  
  
 

| Sequence | MissedCleavage | Mass [Da] | wtIntensity | muIntensity | Score |
| --- | --- | --- | --- | --- | --- |
| AFDAARQER | 1 | 1062.52 | 2954.25 | 5303.56 | 63.08 |
| APGVQTPVIVR | 0 | 1135.67 | 55644.85 | 71910.58 | 202.20 |
| DEEINYFPSR | 0 | 1268.57 | 0.00 | 1180.00 | 114.84 |
| DGMKFPDMVHAFKPNPK | 2 | 1957.95 | 0.00 | 11629.94 | 67.61 |
| EGNFDMVGNNMPVFFIR | 0 | 1985.91 | 14655.99 | 0.00 | 118.48 |
| EKMVIAKENNFMQAGAR | 2 | 1935.97 | 843.69 | 0.00 | 64.84 |
| ENNFMQAGAR | 0 | 1136.50 | 22409.03 | 25668.64 | 214.96 |
| FSTVVHER | 0 | 973.50 | 6593.51 | 0.00 | 114.89 |
| GFFEVTHDISALTAADFLR | 0 | 2109.05 | 11627.57 | 0.00 | 148.44 |
| GPILLEDYHLVEK | 0 | 1524.82 | 59905.08 | 140333.10 | 133.32 |
| GSPETLRDPR | 1 | 1126.57 | 14433.78 | 23409.94 | 70.10 |
| HMPGFGVHTMK | 0 | 1240.58 | 10535.41 | 15936.45 | 108.70 |
| HRLGPNYLLLPVNAPR | 1 | 1829.04 | 0.00 | 1963.06 | 55.72 |
| LAQFDR | 0 | 748.39 | 0.00 | 3053.57 | 103.29 |
| LAQFDRERIPER | 2 | 1528.81 | 1459.11 | 7406.07 | 59.20 |
| LGPNYLLLPVNAPR | 0 | 1535.88 | 25856.79 | 0.00 | 185.14 |
| MVIAKENNFMQAGAR | 1 | 1678.83 | 0.00 | 865.10 | 99.01 |
| VAAVSSAPLSGR | 0 | 1113.61 | 53657.25 | 63732.55 | 170.85 |
| VAAVSSAPLSGRR | 1 | 1269.72 | 47626.90 | 40374.26 | 117.79 |
| VADMLADPR | 0 | 986.49 | 17860.95 | 21844.09 | 166.52 |
| YLLDDEAVMVGGANHSHATK | 0 | 2127.01 | 14418.87 | 26488.57 | 98.17 |
|  |  |  | 360483.0 | 461099.5 |  |
